# Case report: Atypical lipomatous tumor of the thigh in a four-year-old girl

**DOI:** 10.3389/fonc.2024.1401861

**Published:** 2024-07-23

**Authors:** Itaru Ogawa, Michiyuki Hakozaki, Yoichi Kaneuchi, Takeo Suzuki, Takuya Nikaido, Shoki Yamada, Akihito Utsumi, Osamu Hasegawa, Hideki Sano, Yoshihiro Matsumoto

**Affiliations:** ^1^ Department of Orthopaedic Surgery, Fukushima Medical University School of Medicine, Fukushima, Japan; ^2^ Higashi-Shirakawa Orthopaedic Academy, Fukushima Medical University School of Medicine, Fukushima, Japan; ^3^ Department of Diagnostic Pathology, Fukushima Medical University School of Medicine, Fukushima, Japan; ^4^ Department of Radiology, Fukushima Medical University School of Medicine, Fukushima, Japan; ^5^ Department of Pediatric Oncology, Fukushima Medical University Hospital, Fukushima, Japan

**Keywords:** adipocytic tumor, atypical lipomatous tumor, lipoblastoma, child, murine double minute 2, MDM2, cyclin-dependent kinase 4, CDK4

## Abstract

Atypical lipomatous tumors (ALTs) are locally aggressive adipocytic malignancies that frequently occur in middle-aged adults. We report the rare case of an ALT of the thigh that occurred in a 4-year-old girl. Since the tumor was initially diagnosed as a lipoblastoma by incisional biopsy, marginal resection was performed. Histopathological findings of the surgical specimen revealed the proliferation of mature and variously sized adipocytes, as well as ectopic ossification; these features differ from the typical findings of lipoblastoma. Immunohistochemical findings showed nuclear positivity for a murine double minute 2 (MDM2) and cyclin-dependent kinase 4 (CDK4) and negativity for pleomorphic adenoma gene 1 (PLAG1). Fluorescence *in situ* hybridization showed abnormal amplification of the *MDM2* gene. The patient was thus finally diagnosed as having an ALT. No signs of local recurrence or metastasis were noted 1 year postoperatively. This case is instructive in the differential diagnosis of primary adipocytic tumors. Lipoblastomas are the most common adipocytic tumors in children, but if a tumor is located in the deep tissue or imaging findings are not typical, the possibility of ALT should be considered and immunohistochemistry for MDM2 and CDK4 should be added.

## Introduction

Atypical lipomatous tumor (ALT) is an intermediate (locally aggressive) adipocytic tumor that accounts for approx. 40%–45% of all liposarcomas (1). ALT is the most common liposarcoma in middle-aged adults, but it is extremely rare in children, and only 12 cases have been described in children <15 years old (2–9). In these cases, the youngest patient was 5 years old (8). We describe a case that is apparently of the youngest reported patient with an ALT; the tumor developed in the thigh of a 4-year-old girl. The case is also discussed with reference to the relevant literature.

## Case presentation

A previously healthy 4-year-old girl was referred to our hospital with a 1-month history of a soft tissue mass on her right thigh. Her family history was unremarkable for early-onset cancers. On physical examination, an elastic-soft mass (longest diameter. approx. 6.6 cm) was palpable in the anterolateral part of the right proximal thigh. Skin erythema and tenderness were not observed. All of the results of serum biochemical examinations, including liver and renal functions, were within normal range.

**Figure 1:**
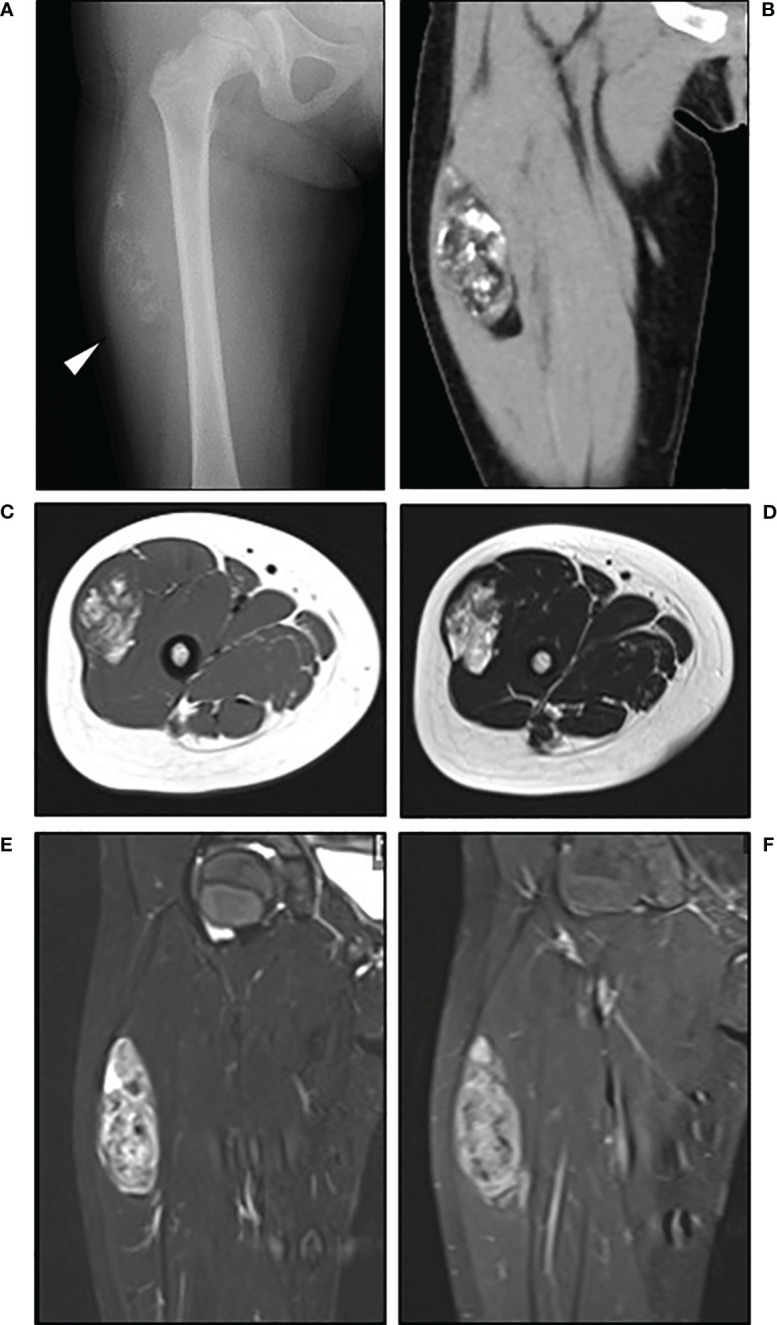
Radiological findings. Plain radiograph **(A)** showed ossification on the anterolateral thigh (*arrow*) of the patient, a 4-year-old girl. Computed tomography [**(B)**, coronal view] revealed a heterogeneous soft tissue mass with fat attenuation that included nodular ossifications. MRI revealed a relatively well-defined tumor in the right vastus lateralis muscle, with heterogeneous high intensity on T1- [**(C)**, axial view] and T2-weighted imaging [**(D)**, axial view], heterogeneous high intensity on short tau inversion recovery imaging [**(E)**, coronal view], and heterogenous enhancement on gadolinium-enhanced T1-weighed fat-suppressed imaging [**(F)**, coronal view].

Plain radiographs showed ossification on the patient’s anterolateral thigh (**Figure 1A**), and computed tomography (CT) revealed a heterogeneous soft tissue mass showing fat attenuation and including multiple nodular ossifications (**Figure 1B**). A relatively well-defined tumor (7.4 × 3.9 × 2.2 cm) was observed in the right vastus lateralis muscle on magnetic resonance imaging (MRI). The tumor generally exhibited high intensity on both T1- and T2-weighted imaging but spotty low-intensity lesions that matched with ossifications were identified on T1- and T2-weighted imaging; heterogeneous high intensity on short tau inversion recovery (STIR) imaging was also observed (**Figures 1C–E**). The tumor had heterogeneous enhancement on gadolinium-enhanced T1-weighed fat-suppression imaging (**Figure 1F**). Although the ossification within the tumor was atypical, adipocytic tumors, particularly lipoblastoma, were initially suspected based on the patient’s age and imaging findings.

An incisional biopsy was performed after the patient’s admission. The histopathological examination detected the proliferation of mature and variously sized adipocytes plus ectopic ossification within the fibrous tissue (which differs from the typical findings of lipoblastoma), but based on her age the patient was diagnosed with lipoblastoma. Then, with the diagnosis of a benign tumor, a marginal resection was planned.

**Figure 2:**
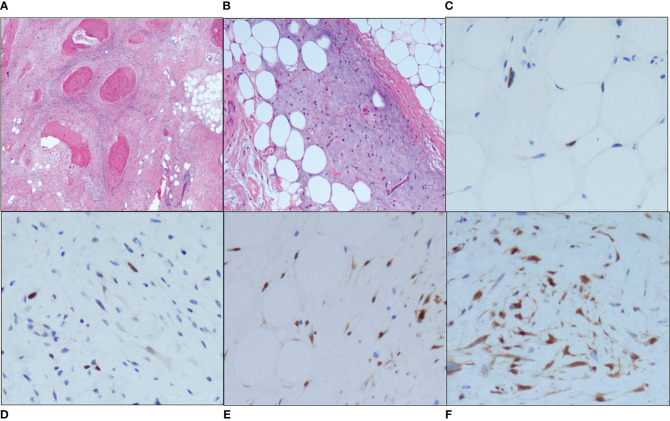
Microscopic findings. **(A)** A low-power view (hematoxylin and eosin [HE] staining, ×40) revealed the proliferation of adipocytes, ossification and surrounding fibrosis. **(B)** A high-power view (HE staining, ×100) showed variously sized adipocytes and atypical stromal cells with nuclear atypia in the myxoid stroma. Both the adipocytes and stromal cells were positive for *MDM2* (×200) [**(C)**, adipocytes; **(D)**, stromal cells] and *CDK4* (×200) [**(E)**, adipocytes; **(F)**, stromal cells].

**Figure 3:**
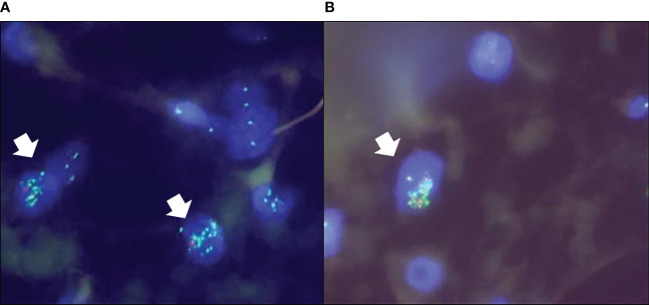
The FISH analysis demonstrated the amplification of the *MDM2* gene in both adipocytes **(A)** and stromal cells **(B)** (*arrows*).

Because of the various immunostainings, the pathological diagnosis required 2 months. After pathological diagnosis, a marginal excision was performed. The histopathologic findings were similar to those of the incisional biopsy, with adipocyte proliferation of various sizes, myxoid stroma, and ossification, but nuclear atypia was slightly more prominent in the stromal cells (**Figures 2A, B**). There were no dedifferentiated components within the tumor. Immunohistochemical staining indicated that the tumor cells were positive for murine double minute 2 (MDM2) (**Figures 2C, D**) and cyclin-dependent kinase 4 (CDK4) (**Figures 2E, F**), and negative for pleomorphic adenoma gene 1 (PLAG1). Fluorescence *in situ* hybridization (FISH) showed abnormal amplification of the *MDM2* gene in adipocytes and stromal cells (**Figure 3**). Based on these findings, the final diagnosis was changed to ALT. Since the surgical margin was pathologically diagnosed as negative (R0), no additional resection was performed. Twelve months after the operation, the patient had no sign of recurrence.

## Discussion

Benign and intermediate (locally aggressive) adipocytic tumors are classified into lipoblastoma, lipoma, and ALT. Among benign tumors, lipoblastomas generally occur in infancy, and they are usually (75%–90%) found during the first 3 years of life (10). Lipoblastomas are seen less frequently in older children and adolescents and rarely in adults (10, 11). Lipomas are the most common soft tissue tumor in adults, with a peak incidence between the fourth and fifth decades of life; they are rare in children (12, 13). ALT, a locally aggressive adipocytic tumor, is the largest subtype of adipocytic malignancies, accounting for approx. 30%–45% of all liposarcomas (1). ALTs occur predominantly in middle-aged adults and are extremely rare in pediatric populations, other than children with Li-Fraumeni syndrome (1, 8, 9, 14). In the present case, a lipoblastoma was strongly suspected initially because she was 4 years old, Li-Fraumeni syndrome had been ruled out, and the pathological findings of an incisional biopsy indicated an adipocytic tumor.

Alternatively, ossifications in the adipocytic tumor were observed in the image findings of the present case. Intertumoral ossifications or calcifications have not been reported in lipoblastoma cases, whereas they have been reported in ALT cases (15). Differential diagnoses of other adipocytic tumors with calcification/ossification include dedifferentiated liposarcoma (DDLPS), osteolipoma, and chondroid lipoma (12, 16, 17). DDLPS is a malignant adipocytic tumor that derives from the dedifferentiation of an ALT, and ossification or calcification is observed in 10-32% of cases on CT scans (17). Unlike ALT, DDLPS is a high-grade and aggressive tumor, with a local recurrence rate of at least 40%, a metastatic rate of 15-30%, and an overall mortality rate of 28-30% (16, 18). Determining whether an ALT has been dedifferentiated into DDLPS is important for the patient’s prognosis. Osteolipoma and chondroid lipoma are rare variants of lipoma in which mature lamellar bone or chondroid are present (12, 19). In addition, fat necrosis should be considered in the differential diagnosis as a non-tumorous lesion. Fat necrosis is a benign, non-suppurative inflammation of the adipose tissue, often accompanied by calcification, and occurs after trauma or surgery (20, 21). Fat necrosis generally occurs in the breast and it is often difficult to distinguish fat necrosis from breast cancer, but it can also occur in the limbs and other parts of the body trunk (21). DDLPS, osteolipoma, chondroid lipoma, and fat necrosis can sometimes be complex and cannot be distinguished from ALT with ossification/calcification by imaging alone. Accurate preoperative diagnosis through biopsy is crucial for establishing the correct treatment plan.

The histopathological findings of our patient’s surgical specimen showed the proliferation of mature and variously sized adipocytes and ectopic ossification, and the immunohistochemical staining indicated that tumor nuclei were positive for MDM2 and CDK4. However, the tumor nuclei were negative for PLAG1, which was reported to have high sensitivity and specificity for lipoblastoma (22). The FISH analysis conducted for the present patient showed abnormal amplification of the *MDM2* gene. The final diagnosis was thus ALT.

As noted above, ALTs in children are extremely rare. Only 12 cases, including our patient, have been described in children <15 years old (**Table 1**). Our patient is the youngest of all of the reported patients. Among the 12 patients, four had genetic changes (e.g., Li-Fraumeni syndrome), and the other eight patients, including the present case, did not have a genetic background. The male/female ratio for the 12 patients is 1:3. The thigh was the predominant site of the pediatric ALTs; seven cases (including our patient’s) occurred in the thigh, which is similar to the scenario in adults. Conversely, ALT of the retroperitoneum, another preferred site in adults, occurred in only one of the 12 children. Generally, lipoblastoma and lipoma are superficial, whereas ALT is deep tissue (11). Of the 7 pediatric ALT cases that occurred in the thigh, 3 previous cases (Case No. 1, 7, 9) and the present cases had a detailed description of the site of origin, and all of which occurred deep in the thigh.

**Table 1:** Previously reported cases of pediatric atypical lipomatous tumor.

Case No.	References	Age, yrs/Sex	Location	Treatment	Prognosis	Immunohistochemistry		MDM2 FISH	Hereditary disease
						CDK4	MDM2		
1	Dadone-Montadié et al. (2)	7/F	Thigh (Adductor muscle)	N/R	N/R	+	N/R	Amplification (+)	–
2	Peng et al. (3)	7/F	Face	Excision	No recurrence	+	+	Amplification (+)	–
3	Özşen et al. (4)	8/F	Thigh	N/R	N/R	+	+	N/R	–
4		10/F	Retroperitoneum	N/R	N/R	–	–	N/R	–
5	Alaggio et al. (5)	15/F	Thigh	Excision	Recurrence at 5yrs	N/R	N/R	N/R	–
6	Kukull et al. (6)	13/F	Gastrocnemius	Excision	Recurrence at 5yrs	+	+	Amplification (+)	–
7	Kuhnen et al. (7)	14/F	Thigh (Posterior compartment)	Excision	N/R	N/R	N/R	Amplification (+)	–
8	Debelenko et al. (8)	5/M	Antecubital fossa	Excision	No recurrence	–	N/R	N/R	Li–Fraumeni syndrome
9		6/M	Thigh (Posterior compartment)	Excision	DOC	–	N/R	N/R	
10	Hammer et al. (9)	8/F	Thigh	Excision	N/R	–	N/R	Amplification (–)	
11		14/M	Subclavicular region	Excision	N/R	–	N/R	Amplification (–)	
12	**Present case**	**4/F**	**Thigh** (Vastus lateralis muscle)	**Excision**	**No recurrence for 1 yr**	**+**	**+**	**Amplification (+)**	**–**

CDK4, cyclin-dependent kinase 4; MDM2, murine double minute 2; FISH, fluorescence in situ hybridization; N/R, not recorded; DOC, died of other cause.

The bold was used to emphasize the present case.

The “+” symbols indicated “Positive”, and the “-” symbols indicated “Negative”.

Concerning the treatment, excision was performed as a local treatment in most of the pediatric ALT cases. Local recurrence of ALT in adults has been reported in 15.1% of cases in the limb and 46.1% in the retroperitoneum (23). The risk of dedifferentiation has been reported in 2%–11.3% of ALTs in the limb and 12.5%–28.5% of ALTs in the retroperitoneum (1, 24). As for surgical therapy, we have two choices: marginal or wide resection. Although the rates of both local recurrence and dedifferentiation were reported to be higher with marginal resection compared to wide resection (17, 24). Especially in younger patients, marginal resection is preferable, because of muscle preservation and maintenance of postoperative mobility. Among the 12 pediatric cases, local recurrence was detected in three of the six cases in which the prognosis was stated, and the youngest case of dedifferentiated liposarcoma was reported in a 12-year-old girl (5). Long-term follow-up is thus mandatory for local recurrence or dedifferentiation.

## Conclusion

We have described an ALT that occurred in a 4-year-old girl. Even in pediatric and adolescent adipocytic tumors, the possibility of locally aggressive or malignant tumors should not be easily ruled out, thus various differential diagnoses should be considered in clinical practice. In particular, if a tumor is located in the deep tissue or imaging findings are not typical, the possibility of ALT should be considered and immunohistochemistry for MDM2 and CDK4 are mandatory.

## Data availability statement

The raw data supporting the conclusions of this article will be made available by the authors, without undue reservation.

## Ethics statement

The requirement of ethical approval was waived by the ethical review committee of Fukushima Medical University for the studies involving humans because at our institution, case reports do not require Ethics Review Committee approval. The studies were conducted in accordance with the local legislation and institutional requirements. Written informed consent for participation in this study was provided by the participants’ legal guardians/next of kin. Written informed consent was obtained from the minor(s)’ legal guardian/next of kin for the publication of any potentially identifiable images or data included in this article.

## Author contributions

IO: Data curation, Writing – original draft. MH: Conceptualization, Funding acquisition, Project administration, Writing – review & editing. YK: Writing – original draft. TS: Writing – original draft. TN: Writing – review & editing. SY: Data curation, Writing – original draft. AU: Data curation, Writing – original draft. OH: Data curation, Writing – original draft. HS: Writing – review & editing. YM: Supervision, Writing – review & editing.
